# Favorable maternal and neonatal outcomes in probable Eisenmenger physiology with suspected DORV: a case report and literature review

**DOI:** 10.1186/s43044-026-00781-0

**Published:** 2026-09-20

**Authors:** Mohamed Mukhtar Mohamed, Mehmet Necmeddin Sutaşır, Khadija Yusuf Ali, Osman Farah Dahir, Nurto Abdulkadir Said, Tuba Doğan, Sharmarke Hassan Ali, Nihat Müjdat Hökenek

**Affiliations:** 1 Department of Emergency Medicine, Mogadishu Somali Turkiye Training and Research Hospital, Mogadishu, Somalia; 2 Faculty of Medicine and Health Sciences, Hormuud University, Mogadishu, Somalia; 3 Department of Emergency Medicine, Başakşehir Cam and Sakura City Hospital, Istanbul, Turkey; 4 Department of Obstetrics and Gynecology, Mogadishu Somali Turkiye Training and Research Hospital, Mogadishu, Somalia; 5 Department of Cardiology, Mogadishu Somali Turkiye Training and Research Hospital, Mogadishu, Somalia; 6 Faculty of Medicine and Health Sciences, Hormuud University, Mogadishu, Somalia; 7 Department of Thoracic Surgery, Mogadishu Somalia Turkiye Training and Research Hospital, Mogadishu, Somalia

**Keywords:** Probable Eisenmenger physiology, Suspected double-outlet right ventricle, Pulmonary hypertension, Vaginal delivery, Maternal outcome, Neonatal outcome, Case report, Literature review, Resource-limited setting

## Abstract

**Background:**

Pregnancy in Eisenmenger syndrome is associated with very high maternal and fetal risk. Complex congenital anatomy further complicates diagnosis and peripartum management, particularly where advanced imaging and pulmonary hypertension therapies are limited. Published evidence specifically addressing pregnancy with uncorrected DORV and Eisenmenger physiology remains sparse.

**Case presentation:**

A 25-year-old primigravida at 34 weeks’ gestation presented with progressive dyspnoea and generalized oedema. The available record documented a heart rate of 130 beats/min, respiratory rate of 25 breaths/min, oxygen saturation of 80% despite face-mask oxygen, a loud second heart sound, and a systolic murmur. Echocardiography showed large ventricular and atrial septal defects with colour Doppler flow, severe tricuspid regurgitation, and an estimated systolic pulmonary artery pressure of 85 mmHg. In the setting of marked hypoxaemia, these findings supported probable Eisenmenger physiology, although shunt direction and pulmonary vascular resistance were not documented. The aorta appeared to arise predominantly from the right ventricle, supporting suspected DORV, but the origin of both great arteries, VSD-great-artery relationship, and DORV subtype could not be defined. Following cardiology and obstetric assessment, labour was induced and vaginal delivery occurred four hours later. The liveborn neonate weighed 2.7 kg, had an initial Apgar score of 5, and was admitted to neonatal intensive care for stabilisation and monitoring. Postpartum haemoglobin fell to 7 g/dL and two units of packed red cells were transfused. The mother was monitored in intensive care for three days, transferred to cardiology on day 6, and discharged in stable condition on day 7.

**Conclusion:**

This report documents maternal survival through induced vaginal delivery and the first postpartum week, with a live birth, despite late recognition of probable Eisenmenger physiology and suspected complex congenital heart disease. The report does not establish the safety of vaginal delivery in Eisenmenger syndrome, and incomplete haemodynamic, anatomical, anaesthetic, and neonatal data limit causal interpretation.

**Supplementary Information:**

The online version contains supplementary material available at https://doi.org/10.1186/s43044-026-00781-0.

## Background

Eisenmenger syndrome is the advanced consequence of an unrepaired systemic-to-pulmonary shunt, in which progressive pulmonary vascular remodelling produces severe pulmonary hypertension and bidirectional or right-to-left shunting [1]. Pregnancy-related increases in blood volume and cardiac output, together with peripartum changes in systemic vascular resistance and venous return, can precipitate hypoxaemia, right ventricular failure, arrhythmia, thrombosis, and death [2]. Maternal and fetal risk remains substantial, particularly around delivery and the early postpartum period [3].

DORV is a heterogeneous congenital cardiac malformation in which both great arteries arise entirely or predominantly from the right ventricle [4]. Physiological consequences depend on the location of the VSD, the relationship of the great arteries, and the presence of pulmonary outflow obstruction. Precise anatomical classification therefore requires clear demonstration of both great arteries and their relationship to the VSD [5].

We report the short-term maternal and neonatal outcomes of a woman whose first cardiac assessment occurred late in pregnancy and who underwent induced vaginal delivery in a resource-limited hospital. The clinical question is not whether vaginal delivery is generally safe in Eisenmenger syndrome, but how this individual patient survived delivery and the immediate postpartum period despite probable Eisenmenger physiology, suspected complex congenital anatomy, and limited diagnostic and therapeutic resources.

## Methods of data collection and literature review

This case report was prepared by retrospectively reviewing the available contemporaneous admission documentation, including emergency, cardiology, obstetric, intensive-care, laboratory, electrocardiographic, chest radiographic, transthoracic echocardiographic, neonatal-summary, and discharge information. Data were cross-checked against the available images and discharge documentation. No additional investigation was performed for research purposes. Where a clinical detail was absent from the available record, it is reported as unavailable rather than inferred.

A focused narrative literature search was conducted in PubMed/MEDLINE through 30 July 2026 using the terms “Eisenmenger syndrome” AND “pregnancy”, “double-outlet right ventricle” AND “pregnancy”, “DORV” AND “pregnancy”, and “pulmonary arterial hypertension” AND “pregnancy”. Reference lists of relevant articles were screened for additional reports. Current guidelines, observational studies, case series, and case reports directly addressing pregnancy in Eisenmenger syndrome or DORV were included. Because the evidence was sparse and clinically heterogeneous, findings were synthesised narratively; this was not a systematic review and no meta-analysis was performed. Three Somali publications were included to provide local context: a study of peripartum cardiomyopathy, a case report of cor triatriatum dextrum with an atrial septal defect and pulmonary hypertension in pregnancy, and an intensive-care study of pre-eclampsia/eclampsia. The cor triatriatum report was treated as a congenital-heart-disease comparator, whereas the two observational studies were used only to contextualise the broader local burden of severe pregnancy-associated cardiac and maternal illness; none was treated as direct evidence about Eisenmenger syndrome or DORV.

## Case presentation

A 25-year-old woman, gravida 1 para 0, from a rural area presented at 34 weeks’ gestation with progressive shortness of breath and generalized oedema over several days. She had no prior cardiac evaluation or childhood screening for congenital heart disease. A detailed childhood and pre-pregnancy functional history, including exercise intolerance, easy fatigability, recurrent respiratory infections, squatting, syncope, and longstanding cyanosis, was not documented; absence of these features therefore cannot be assumed.

The available examination record documented a heart rate of 130 beats/min, a respiratory rate of 25 breaths/min, oxygen saturation of 80% despite supplemental oxygen by face mask, a loud second heart sound, and a systolic murmur. Blood pressure was recorded as within normal limits. The record referred to peripheral cyanosis and volume overload but did not provide sufficiently reliable detail to determine the presence of central cyanosis, clubbing, raised jugular venous pressure, pulmonary crackles, or the precise distribution of oedema.

**Fig. 1:**
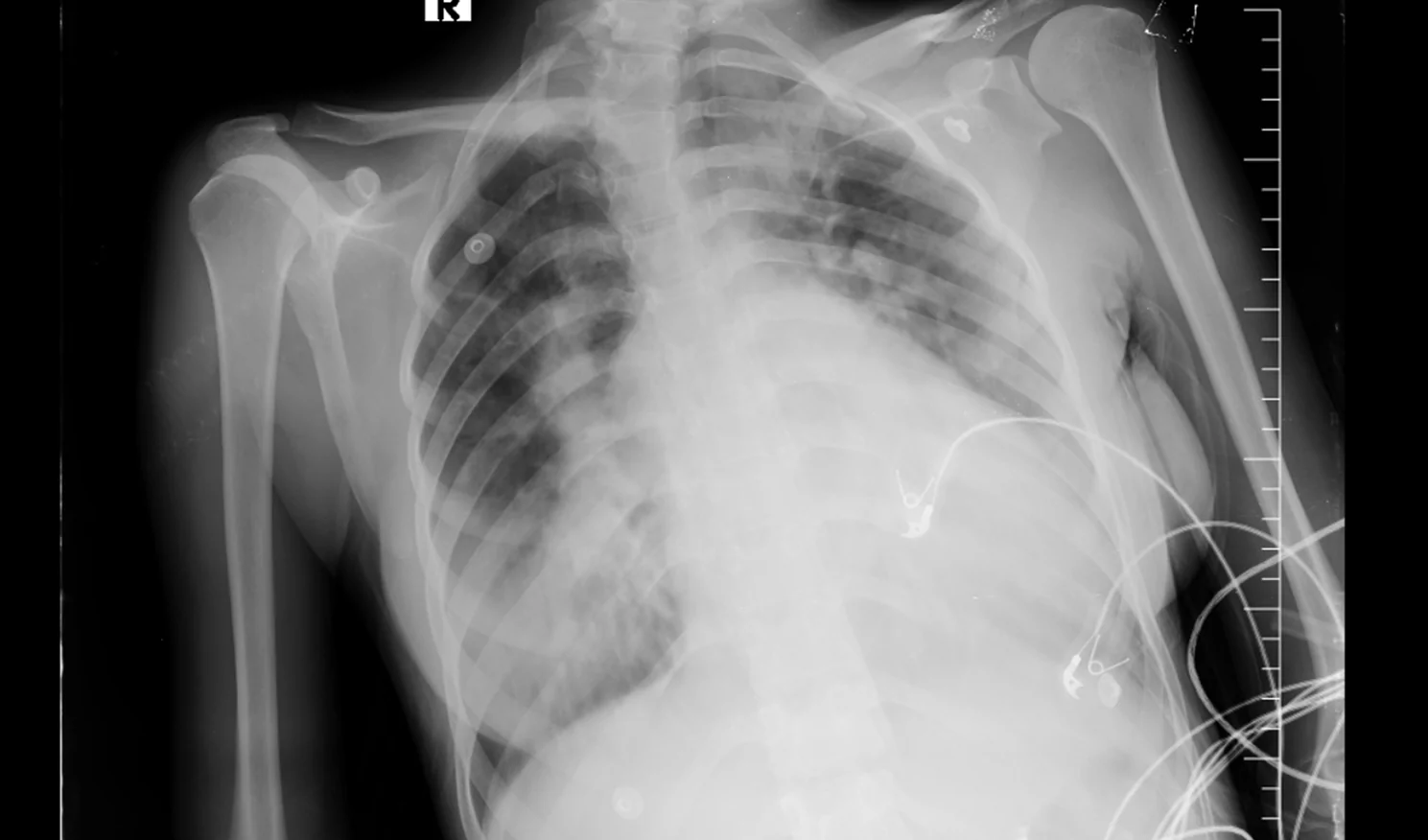
Chest radiograph showing cardiomegaly and bilateral pulmonary vascular/interstitial congestion

**Fig. 2:**
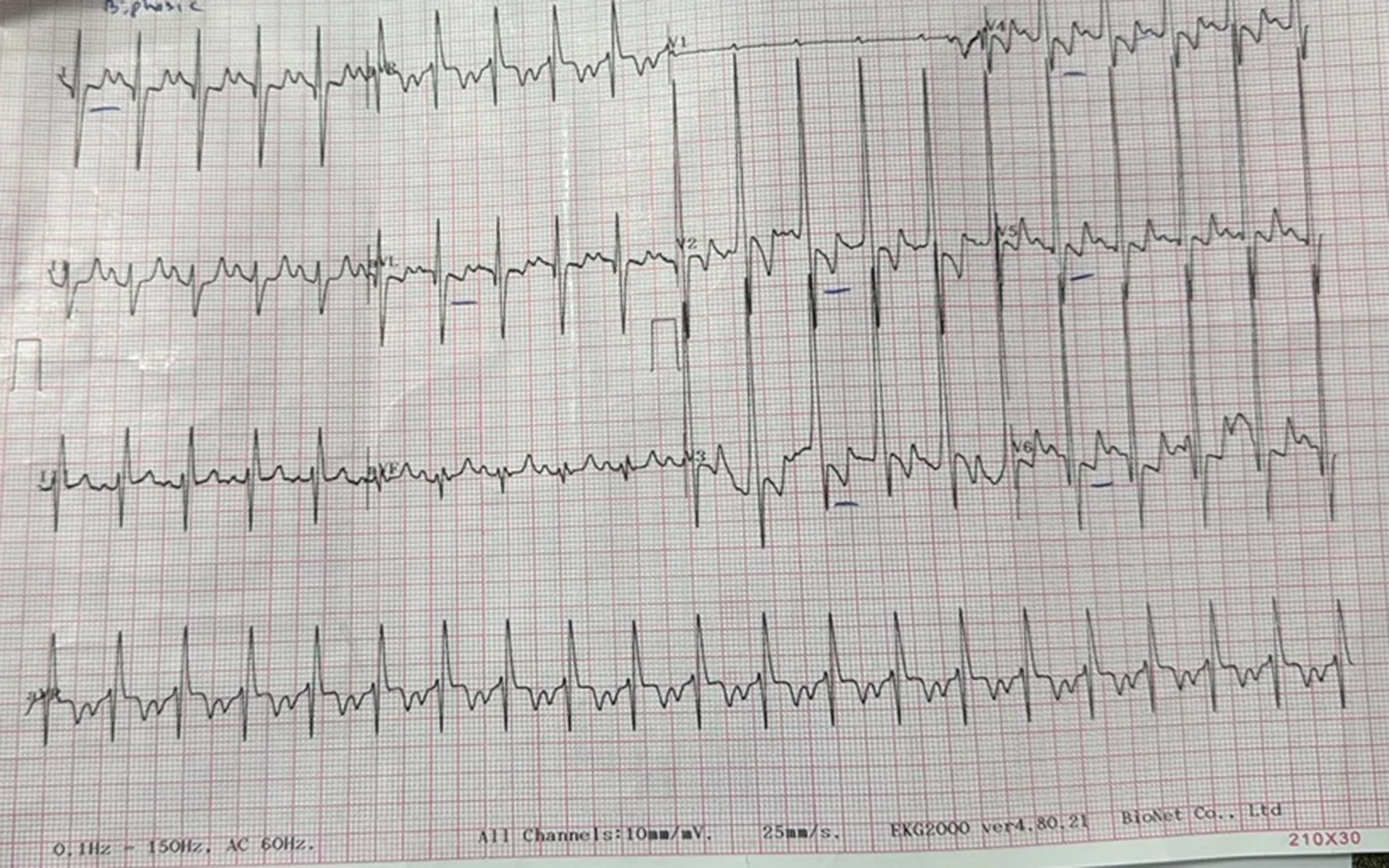
Electrocardiogram showing sinus tachycardia with features of right ventricular strain

Laboratory investigations showed haemoglobin 8.2 g/dL, haematocrit 29.4%, white-cell count 10.75 × 10^3/mm3, and C-reactive protein 110 mg/L. Renal, hepatic, and thyroid function tests were within the local reference ranges; HIV, hepatitis B, and hepatitis C serology was negative. Chest radiography showed cardiomegaly and bilateral pulmonary oedema (Fig. 1), and electrocardiography showed sinus tachycardia with a right ventricular strain pattern (Fig. 2).

**Fig. 3:**
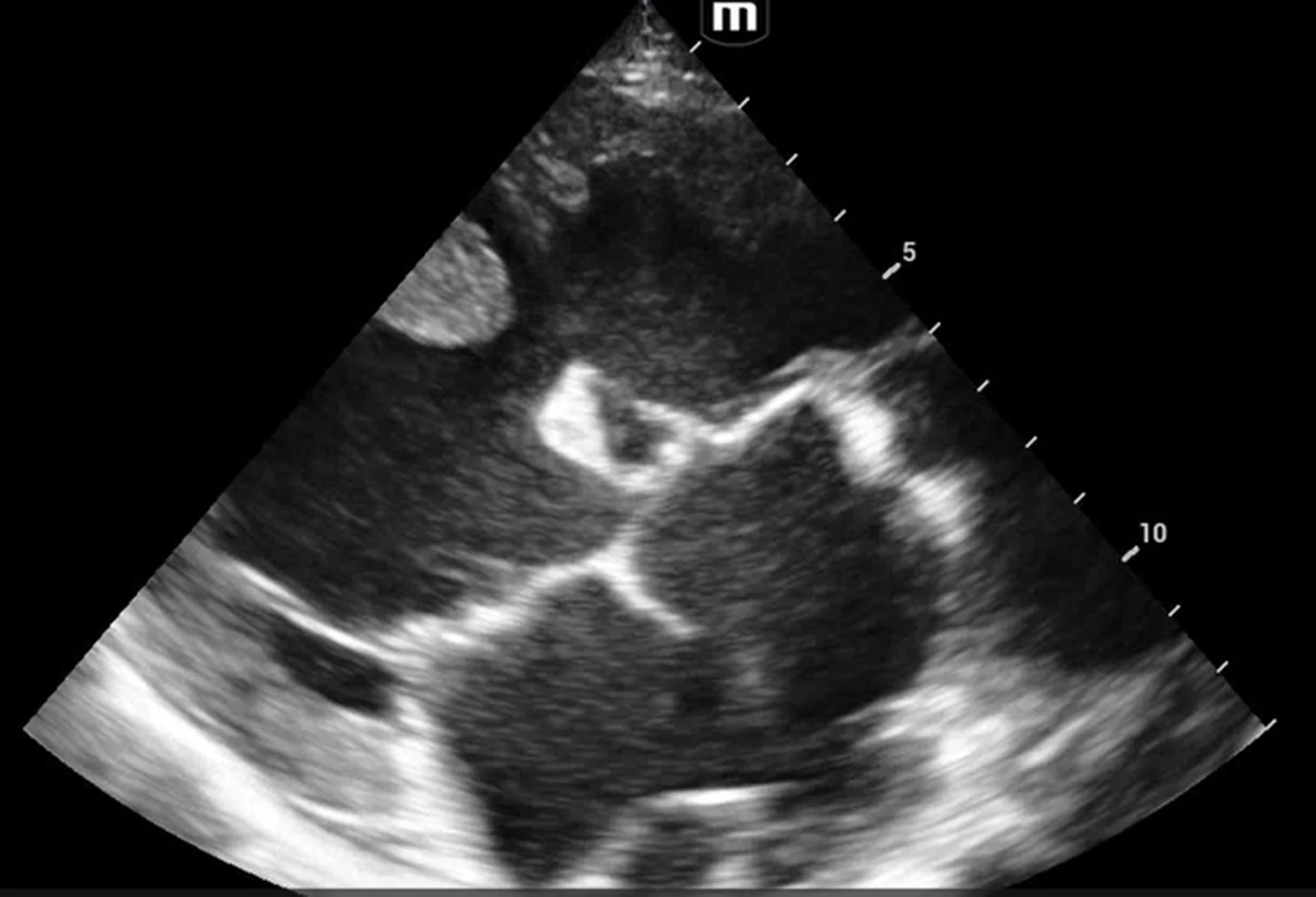
Modified four-chamber transthoracic echocardiographic view demonstrating large ventricular and atrial septal defects

**Fig. 4:**
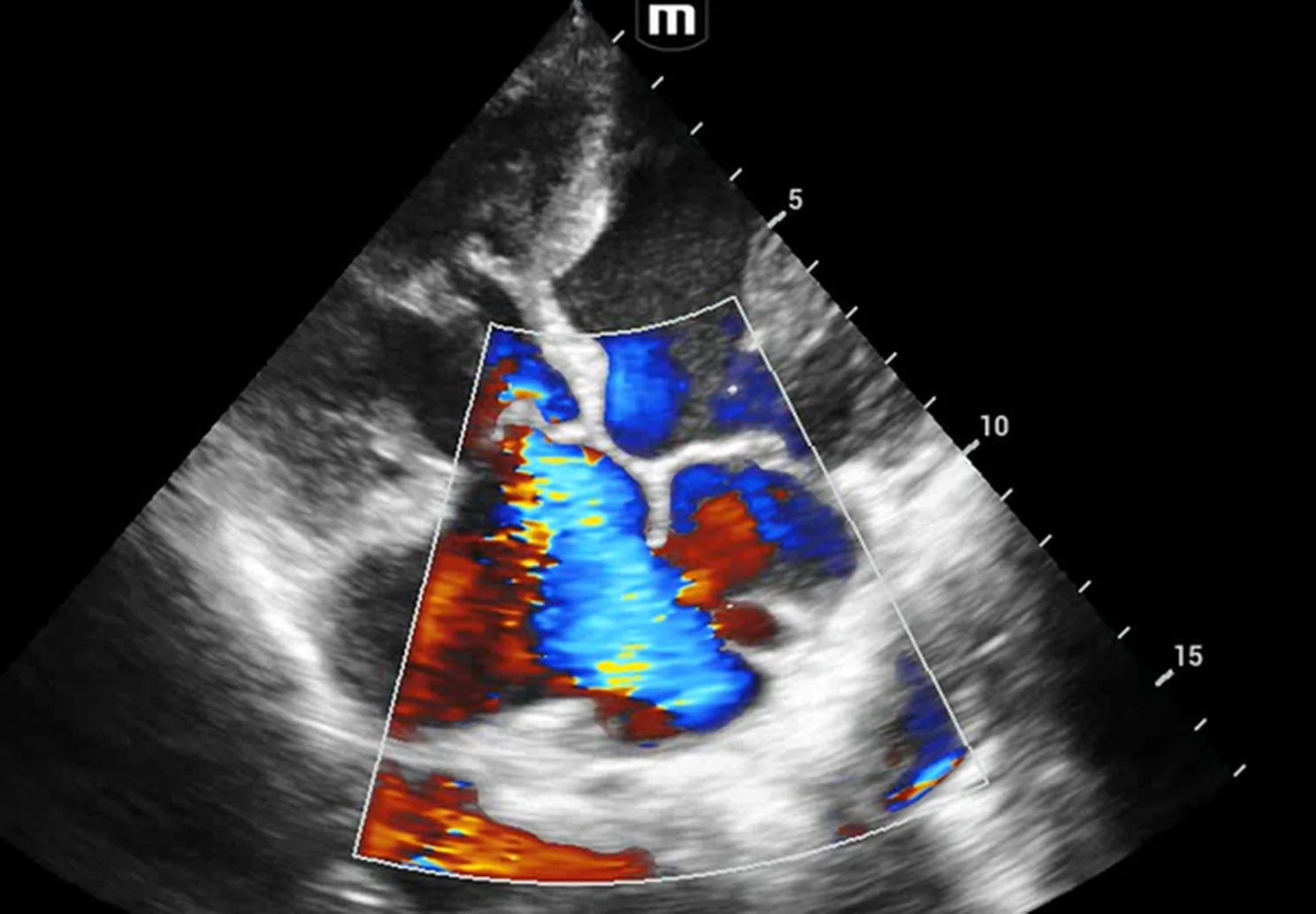
Colour Doppler transthoracic echocardiography demonstrating intracardiac flow and severe tricuspid regurgitation; estimated systolic pulmonary artery pressure was approximately 85 mmHg

**Fig. 5:**
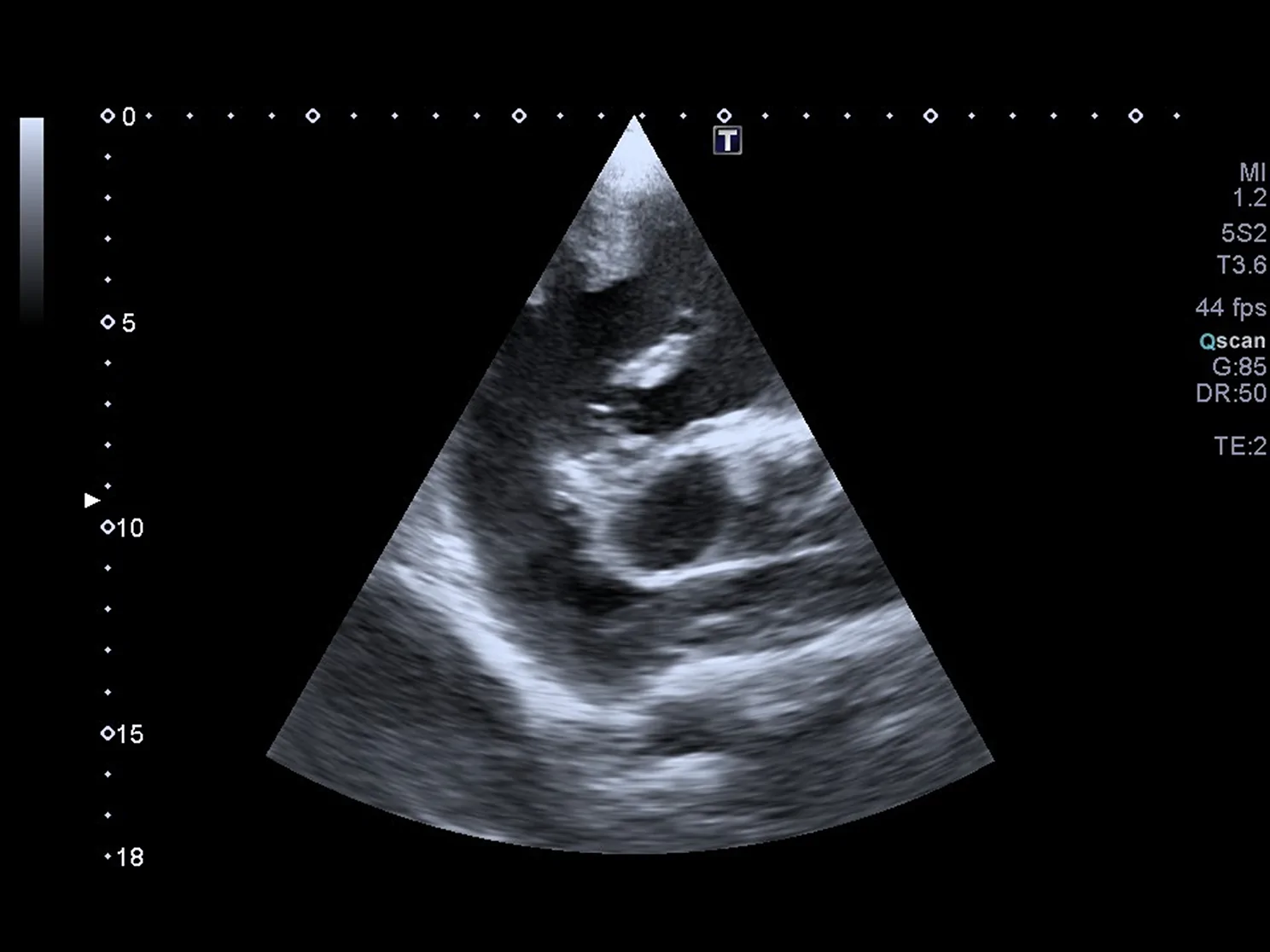
Echocardiographic view in which the aorta appears to arise predominantly from the right ventricle. The image supports suspected DORV but does not, by itself, demonstrate the origin of both great arteries or define the VSD-great-artery relationship. No better archived still frame or cine loop could be retrieved

Transthoracic echocardiography demonstrated a large VSD and a large ASD (Fig. 3) with demonstrable colour Doppler flow, severe tricuspid regurgitation, and an estimated systolic pulmonary artery pressure of approximately 85 mmHg (Fig. 4). The direction of flow across the septal defects was not documented. In the setting of marked hypoxaemia and cyanosis, the findings were considered suggestive of probable Eisenmenger physiology, but the absence of documented bidirectional or right-to-left shunting and the lack of right-heart catheterisation prevent definitive confirmation. The aorta appeared to arise predominantly from the right ventricle, supporting suspected DORV (Fig. 5); however, no available still frame or cine loop clearly demonstrated the origin of both great arteries or defined the VSD-great-artery relationship. The VSD could therefore not be classified as subaortic, subpulmonary, doubly committed, or remote. Left ventricular systolic function and quantitative right ventricular function were not recorded. No better archived echocardiographic image could be retrieved.

Following evaluation by the cardiology and obstetric teams, labour was induced after the patient and family were counselled regarding maternal and fetal risk. The available record did not identify the induction agent or Bishop score and did not document participation by anaesthesia or neonatal teams, the anaesthetic or analgesic technique, fetal-monitoring method, individual labour-stage durations, use of assisted second stage, estimated blood loss, invasive haemodynamic monitoring, or the detailed fluid-management strategy.

Vaginal delivery occurred four hours after induction. The liveborn neonate weighed 2.7 kg, had an initial Apgar score of 5, and was admitted to the neonatal intensive care unit for stabilisation and monitoring. The available record did not specify a five-minute Apgar score, resuscitation measures, respiratory support, neonatal echocardiographic findings, duration of neonatal admission, or condition at neonatal discharge. The neonatal outcome is therefore limited to live birth and initial stabilisation.

Postpartum care included supplemental oxygen, diuretics for congestion, and antibiotics in the context of raised inflammatory markers. On postpartum day 2, oxygen saturation remained low and haemoglobin had fallen from 8.2 g/dL before delivery to 7 g/dL. Two units of packed red cells were transfused slowly. She remained under ICU monitoring for three days, was transferred to the cardiology inpatient service on day 6, and was discharged in stable condition on day 7. The available record did not document pulmonary vasodilator therapy, vasopressors, inotropes, non-invasive ventilation, or invasive mechanical ventilation; this is absence of documentation rather than proof that any intervention was never considered. Contraception counselling or a selected method was not documented.

## Discussion

### Literature review and comparison with published evidence

Historical evidence demonstrates why survival in an individual case should not be equated with safety. A systematic overview of 73 pregnancies complicated by Eisenmenger syndrome reported maternal mortality of 36%; apart from three antepartum deaths, all fatalities occurred during delivery or within 35 days postpartum [6].

A West China series of 11 pregnancies reported four maternal deaths, giving the same mortality rate of 36%. Adverse fetal outcomes were also frequent: preterm delivery occurred in 88%, small-for-gestational-age birth in 83%, fetal death in 27%, and neonatal death in 25% [7].

In a Japanese series of 15 women, only five continued pregnancy. Those five developed worsening cyanosis, exertional fatigue, and dyspnoea between 25 and 30 weeks and underwent preterm caesarean delivery at a median of 30 weeks, illustrating that clinical deterioration may emerge before the third trimester is complete [8].

A retrospective cohort from Indonesia described 18 pregnancies with Eisenmenger syndrome, of which seven ended in maternal death from cardiogenic shock. Preterm birth occurred in 78%, low birth weight in 94%, and fetal growth restriction in 55%, underscoring the combined maternal and placental-fetal burden in a lower-resource setting [9].

A 2023 case series and literature review identified 72 pregnancies across 11 studies. Pulmonary hypertension-targeted therapy was documented in only 28%, while perinatal maternal mortality remained 24%. These observational data suggest possible benefit from specialist disease-targeted management but cannot establish treatment efficacy because of selection bias, inconsistent regimens, and the absence of controlled comparisons [10].

A Northern Indian series included 12 pregnancies in 11 women; approximately 80% were diagnosed during pregnancy. Preeclampsia occurred in 50%, fetal growth restriction in 62.5%, and three women died in the postpartum period, corresponding to maternal mortality of 27% [11].

A prospective tertiary-centre cohort comparing 30 pregnancies with pulmonary arterial hypertension and 20 with Eisenmenger syndrome further illustrates the heterogeneity of anaesthetic, medical, and obstetric management. The study supports multidisciplinary, individualised planning rather than a single delivery strategy for all patients [12].

Published experience with uncorrected DORV in pregnancy is much more limited. Gu and colleagues reported a 28-year-old woman presenting at 30 weeks with NYHA class III-IV symptoms and uncorrected DORV. She underwent caesarean delivery under epidural anaesthesia with phenylephrine support, experienced episodes of dyspnoea and desaturation, and was discharged on postoperative day 7; the premature neonate required intensive care [13].

Tilak and Wankhede described a 30-year-old woman in whom uncorrected DORV with VSD was first recognised at 22 weeks after severe breathlessness and recurrent haemoptysis. Their report emphasised early diagnosis and stepwise multidisciplinary management but did not address Eisenmenger physiology in the same detail as the present case [14].

A separate report documented successful pregnancy in a 19-year-old woman with DORV but without haemodynamic or structural complications and without fetal abnormality. That substantially lower-risk physiology limits direct comparison with a cyanotic patient with severe pulmonary hypertension [15].

Another uncorrected DORV report described two previous fetal losses followed by a pregnancy complicated by thrombocytopenia, placenta praevia, and severe fetal growth restriction. Emergency caesarean delivery was followed by postpartum haemorrhage and COVID-19 infection, demonstrating that obstetric and non-cardiac complications can compound the underlying congenital lesion [16].

The 2025 European Society of Cardiology pregnancy guideline places Eisenmenger syndrome in the highest maternal-risk category and emphasises comprehensive counselling, shared decision-making, and care by a Pregnancy Heart Team when pregnancy continues. Even with contemporary treatment, reported maternal mortality in pulmonary arterial hypertension remains approximately 11%-25% [17].

A contemporary single-centre study of 42 parturient women with Eisenmenger syndrome likewise reported high maternal and neonatal risk, frequent preterm birth, and low birth weight. The authors advised against pregnancy and stressed that late-pregnancy continuation requires explicit discussion of the risk of maternal death [18].

Highly specialised rescue strategies have occasionally supported survival. One report used a novel extracorporeal membrane oxygenation configuration as a bridge to delivery and recovery, but such resource-intensive rescue cannot be generalised to hospitals without advanced mechanical circulatory support [19].

Current pulmonary hypertension guidance recommends expert-centre management and specialist selection of pregnancy-compatible pulmonary arterial hypertension therapy when pregnancy continues. Evidence for treatment during pregnancy remains observational, and several pulmonary vasodilators, particularly endothelin-receptor antagonists, are contraindicated because of fetal toxicity [20].

A recent retrospective study from Somalia reported 20.2% mortality among 72 women with peripartum cardiomyopathy. Although peripartum cardiomyopathy is pathophysiologically distinct from Eisenmenger physiology, this finding provides local context for the broader burden of pregnancy-associated cardiac disease and the need for earlier recognition and specialist multidisciplinary care in resource-limited settings [21].

An earlier Somali case report described a 35-year-old woman with previously unrecognised cor triatriatum dextrum, a primum atrial septal defect, and pulmonary hypertension who presented early in pregnancy with dyspnoea, palpitations, and haemoptysis. After treatment with oxygen, furosemide, digoxin, and enoxaparin, she later delivered a healthy term infant vaginally under non-invasive monitoring [22]. Although the anatomy and haemodynamics differ from probable Eisenmenger physiology with suspected DORV, this report provides a useful local comparator: pregnancy may reveal previously unrecognised congenital heart disease, and accurate echocardiographic definition with longitudinal multidisciplinary follow-up remains important.

Severe maternal illness requiring critical care is also a recognised local challenge. In a retrospective Mogadishu cohort of 71 women with pre-eclampsia/eclampsia admitted to intensive care, 91.5% required mechanical ventilation and in-hospital mortality was 11.1% [23]. This cohort concerned hypertensive disorders rather than congenital heart disease and is therefore cited only to contextualise the critical-care demands and maternal risk in the same resource-limited setting.

### Interpretation of the present case

The novel observation in this case is the short-term outcome: a woman with previously unrecognised cyanotic congenital heart disease, severe pulmonary hypertension, probable Eisenmenger physiology, and suspected DORV survived induced vaginal delivery and the first postpartum week, and delivered a liveborn infant who underwent initial neonatal stabilisation. This outcome is clinically notable, but it must not be interpreted as evidence that pregnancy or vaginal delivery is safe in Eisenmenger syndrome.

Compared with the published DORV reports, this case is unusual because marked hypoxaemia and severe pulmonary hypertension suggested probable Eisenmenger physiology, while the available image supported only suspected DORV, and vaginal delivery occurred in a resource-limited setting. The report adds one short-term observation to a very small and heterogeneous literature; it neither confirms the anatomical diagnosis nor permits comparison of vaginal and caesarean delivery.

Mode of delivery should be individualised. Vaginal delivery can avoid surgical blood loss, infection, and abrupt haemodynamic effects of anaesthesia, but pain, Valsalva manoeuvres, hypotension, and postpartum autotransfusion can also provoke decompensation. In this case, cardiology and obstetric assessment, a four-hour induction-to-delivery interval, and postpartum access to intensive care were documented. Because the anaesthetic, monitoring, second-stage, blood-loss, and fluid-management details were unavailable, the contribution of specific peripartum interventions cannot be assessed.

The postpartum period is particularly hazardous because autotransfusion from the contracting uterus and rapid fluid redistribution can increase right ventricular preload while falls in systemic vascular resistance can worsen right-to-left shunting. In this case, documented care included oxygen, diuretics, antibiotics, transfusion of two units of packed red cells after haemoglobin fell to 7 g/dL, three days of ICU monitoring, transfer to cardiology on day 6, and discharge in stable condition on day 7. No pulmonary vasodilator, vasopressor, inotrope, non-invasive ventilation, or invasive ventilation was documented; the report therefore cannot attribute stability to these therapies or conclude that they were unnecessary.

The cardiac diagnosis requires particular caution. Marked hypoxaemia, cyanosis, large septal defects, and an echocardiographic pulmonary pressure estimate of 85 mmHg support probable Eisenmenger physiology, but the shunt direction was not recorded and right-heart catheterisation was not performed. Likewise, the apparent aortic origin from the right ventricle supports suspected DORV, yet the available image does not establish that both great arteries arise predominantly from the right ventricle or permit anatomical classification.

The neonatal result should also be described narrowly. A 2.7-kg liveborn infant with an initial Apgar score of 5 was admitted to neonatal intensive care. Absent five-minute Apgar, treatment, discharge, and follow-up data preclude the claim of an uncomplicated or fully favourable neonatal course.

Future pregnancy would carry an unacceptably high risk if Eisenmenger physiology is confirmed. Current guidance supports explicit counselling to avoid further pregnancy and shared specialist selection of an effective contraceptive method, taking account of thrombosis risk, haemodynamic tolerance, local availability, and the patient’s preferences. The available record did not confirm that such counselling occurred in this admission.

## Conclusion

This case reports maternal survival through induced vaginal delivery and the first postpartum week, together with live birth and initial neonatal stabilisation, after late recognition of probable Eisenmenger physiology with suspected DORV. The literature review demonstrates that contemporary survival in selected reports has not removed the substantial maternal and perinatal risk and that pregnancy evidence specific to uncorrected DORV remains limited to small case-based reports. This individual short-term outcome must not be generalised. Future pregnancy should be strongly discouraged if Eisenmenger physiology is confirmed, and effective contraception should be addressed through specialist counselling.

## Limitations

This report is limited by retrospective reliance on incomplete records; absence of a detailed pre-pregnancy symptom history; lack of documented shunt direction or right-heart catheterisation; incomplete echocardiographic definition of both great-artery origins, DORV subtype, VSD relationship, LV function, and quantitative RV function; lack of a better archived echocardiographic image; incomplete documentation of induction method, anaesthesia, analgesia, fetal monitoring, labour stages, blood loss, invasive haemodynamic monitoring, and exact fluid balance; and limited neonatal treatment, discharge, and longer-term follow-up data. These limitations prevent definitive confirmation of Eisenmenger syndrome, definitive anatomical classification of DORV, full attribution of the short-term maternal course to specific interventions, or generalisation of the delivery strategy. The literature review was narrative rather than systematic, was not prospectively registered, and is vulnerable to publication bias, particularly preferential publication of successful DORV pregnancies.

## Supplementary Information

Below is the link to the electronic supplementary material.

Supplementary Material 1

## Data Availability

All relevant data of this manuscript and supporting findings of this study are available from the corresponding author upon reasonable request.

## Abbreviations

ASD: Atrial septal defect
DORV: Double-outlet right ventricle
ES: Eisenmenger syndrome
ICU: Intensive care unit
LV: Left ventricle
RV: Right ventricle
VSD: Ventricular septal defect
