# Supplementary material for: Favorable maternal and neonatal outcomes in probable Eisenmenger physiology with suspected DORV: a case report and literature review
Source: Egypt Heart J. 2026 Sep 20;78:70. doi: 10.1186/s43044-026-00781-0 (PMC13590458; doi:10.1186/s43044-026-00781-0)
Supplement: Supplementary file 1 [file 43044_2026_781_MOESM1_ESM.docx]

CARE Checklist:

| Item No. | Item | **Reported in Manuscript** |
| --- | --- | --- |
| 1 | Title | Title, page 1 |
| 2 | Keywords | Keywords section, page 1 |
| 3 | Abstract | Abstract, 1 |
| 4 | Introduction | Background section, paragraphs 1–4, page 2 |
| 5 | Patient information | Case Presentation, paragraphs 1–2, page 3 |
| 6 | Clinical findings | Case Presentation, paragraph 3, page 3 |
| 7 | Timeline | Case Presentation (overall clinical course), page 3&4 |
| 8 | Diagnostic assessment | Case Presentation, paragraphs 4–5, page 3 |
| 9 | Therapeutic intervention | Case Presentation, paragraphs 6–8, page 3 & 4 |
| 10 | Follow-up and outcomes | N/A |
| 11 | Discussion | Discussion section, page 4 |
| 12 | Patient perspective | N/A |
| 13 | Informed consent | Additional Information – Consent for publication, Page 5 |
